# Kokeshi phenomenon and coronary perforation with rotational atherectomy while treating heavily calcified coronary artery disease: A case report: Retraction

**DOI:** 10.1097/MD.0000000000044336

**Published:** 2025-08-22

**Authors:** Syed Mohammad Naqvi, Syed Yaseen Naqvi, Muhammad Anis Haider, Hashim Talib Hashim, Muhammad Usman Shah, Manish Ramlall, Zaid Saad Abed Madhi, Ashraf Fhed Mohammed Basalilah, Ali Ali

The article “Kokeshi phenomenon and coronary perforation with rotational atherectomy while treating heavily calcified coronary artery disease: A case report”^1^, which appears in Volume 104, Issue 17 of *Medicine*, is being retracted. Information brought to our attention during a recent investigation casts doubt on both the authorship and credibility of the presented case, and the Publisher has lost faith in the integrity of the content.
